# Report of a Case of Umbilical Calculus

**Published:** 1859-07

**Authors:** S. H. Dean

**Affiliations:** Conyers, Georgia


### ARTICLE VII.

*Report of a Case of Umbilical Calculus.* By S. H. DEAN, M. D., of Conyers, Georgia.

On the 29th of May, 1855, I was called to see Mrs. H., who was suffering from an abdominal abscess. Mrs. H. was about 35 years of age, and the mother of several children; the youngest being at the breast.

Several months before this time, the patient had an abscess to form in and about the umbilicus. A physician was called, the abscess was opened, and a large quantity of pus was discharged. Pus continued to be discharged for several weeks, if not for months, but finally ceased, and the abscess healed up. But after a few weeks, another formed in the same place, and I was called to see the patient.

There was, at that time, a great deal of pain, redness and swelling, for several inches around the umbilicus.

Ordered emollient poultices to be applied to the abscess, and anodynes to be used, so as to relieve pain and procure rest.

31st May.—Some superficial sloughing had taken place, about the opening of the abscess, beneath which was a dark body, which had the appearance of a deep slough. But when it was examined with a probe, it was found to be firm and unyielding. I pronounced it an umbilical calculus.—Dr. T. C. H. Wilson was invited to see the case with me, and he confirmed my diagnosis.

We enlarged the opening of the abscess, and removed the stone with forceps. The wound soon healed, and the patient was discharged.

The calculus was of the mulberry variety, and about the size of a partridge egg. It seemed to have been imbedded in a fibrous sac, just below the umbilicus.

The cause of the abscesses was very obscure, until the calculus was discovered.

An interesting inquiry arises, as to the manner in which the stone was formed in that position. There is but one reasonable way of accounting for it—that is, by the supposition that the urachus was an open tube, communicating with the bladder. That a nucleus was formed at or near the umbilicus, and gradually increased in size by deposition from the urine, until it became a source of irritation and inflammation.

Anatomists are disagreed, as to the structure of the urachus. Some say that it is a fibrous cord in fœtal as well as in the adult life—that it is only a ligament of the bladder.—Others say, that in the fœtus, it is an open tube. The probability is, that in the embryo, and in early fœtal life, it is an open tube, and conveys the secretions of the corpora wolfianna and kidneys, into the allantois. But when the uro-genital organs are sufficiently formed, this function is performed by them, and the walls of the urachus, usually, collapse and form a fibrous cord, but sometimes remain open.

Cases of umbilical calculi are very rare. I never have seen but one case, besides the one reported above. That was in a man, operated upon by Dr. Pancoast, of Philadelphia. Three calculi, each about the size of a pea, were removed from a point just below the umbilicus.

